# Prospective early warning signals to detect transitions to manic and depressive episodes in bipolar disorder

**DOI:** 10.1192/j.eurpsy.2021.237

**Published:** 2021-08-13

**Authors:** F. Bos, M. Schreuder, B. Doornbos, E. Snippe, R. Bruggeman, L. Van Der Krieke, B. Haarman, M. Wichers, S. George

**Affiliations:** 1 Department Of Psychiatry, University of Groningen, University Medical Center Groningen, Groningen, Netherlands; 2 Psychiatry, University Medical Center Groningen, Groningen, Netherlands; 3 Bipolar Disorders, GGZ Drenthe, Assen, Netherlands

**Keywords:** prediction, bipolar disorder, early warning signals, experience sampling methodology

## Abstract

**Introduction:**

For patients with bipolar disorder, early recognition of impending mood episodes is crucial to enable timely intervention. Longitudinal digital mood monitoring using ecological momentary assessment (EMA) enable prospective study of early warning signals (EWS) in momentary affective estates prior to symptom transitions.

**Objectives:**

The present study examined in a unique longitudinal EMA data set whether EWS prospectively signal transitions to manic or depressive episodes.

**Methods:**

Twenty bipolar type I/II patients completed EMA questionnaires five times a day for four months (average 491 observations per person), as well as weekly symptom questionnaires concerning depressive (Quick Inventory for Depressive Symptomatology) and manic (Altman Self-Rating Mania Scale) symptoms. Weekly data was used to determine transitions (i.e., abrupt increase in symptoms). Prior to these transitions, EWS (autocorrelation at lag-1 and standard deviation) were calculated in moving windows over 17 affective EMA states. Kendall’s tau was calculated to detect significant rises in the EWS indicator prior to the transition.

**Results:**

Eleven patients reported one or two transitions to a mood episode. All transitions were preceded by at least one EWS. Average sensitivity for detecting EWS was slightly higher for manic episodes (36%) than for depressive episodes (25%). For manic episodes, EWS in thoughts racing, being full of ideas, and feeling agitated showed the highest sensitivity and specificity, whereas for depression, only feeling tired showed high sensitivity and specify.

**Conclusions:**

EWS show promise in anticipating transitions to mood episodes in bipolar disorder. Further investigation is warranted.

**Disclosure:**

No significant relationships.

